# Head-Of-Bed Elevation (HOBE) for Improving Positional Obstructive Sleep Apnea (POSA): An Experimental Study

**DOI:** 10.3390/jcm11195620

**Published:** 2022-09-23

**Authors:** Giannicola Iannella, Giovanni Cammaroto, Giuseppe Meccariello, Angelo Cannavicci, Riccardo Gobbi, Jerome Rene Lechien, Christian Calvo-Henríquez, Ahmed Bahgat, Giuseppe Di Prinzio, Luca Cerritelli, Antonino Maniaci, Salvatore Cocuzza, Antonella Polimeni, Giuseppe Magliulo, Antonio Greco, Marco de Vincentiis, Massimo Ralli, Annalisa Pace, Roberta Polimeni, Federica Lo Re, Laura Morciano, Antonio Moffa, Manuele Casale, Claudio Vicini

**Affiliations:** 1 Department of ‘Organi di Senso’, University “Sapienza”, Viale dell’Università, 33, 00185 Rome, Italy; 2 Department of Head-Neck Surgery, Otolaryngology, Head-Neck and Oral Surgery Unit, Morgagni Pierantoni Hospital, Via Carlo Forlanini 34, 47121 Forlì, Italy; 3 Laboratory of Anatomy and Cell Biology, Faculty of Medicine, University of Mons (UMONS), Avenue du Champ de Mars 6, B7000 Mons, Belgium; 4 Clinic of Otolaryngology, Hospital Complex of Santiago de Compostela, 15706 Santiago de Compostela, Spain; 5 Department of Otorhinolaryngology, Alexandria University, Elazaritta, Alexandria 0020, Egypt; 6 Department ENT & Audiology, University of Ferrara, Via Savonarola 9, 44121 Ferrara, Italy; 7 Department of Medical and Surgical Sciences and Advanced Technologies “GF Ingrassia”, ENT Section, University of Catania, Via S. Sofia 78, 95125 Catania, Italy; 8 Department of Oral and Maxillo Facial Sciences, “Sapienza” University of Rome, 00161 Rome, Italy; 9 School of Medicine, Campus Bio-Medico University, Via Alvaro del Portillo 21, 00128 Rome, Italy

**Keywords:** obstructive sleep apnea, positional obstructive sleep apnea, snoring

## Abstract

Purpose: Evaluate the effectiveness of the head-of-bed elevation position (HOBE) with a 30° elevation of the head and trunk, in improving obstruction of the upper airways in obstructive sleep apnea (OSA) patients. A prospective trial simultaneously performing drug-induced sleep endoscopy (DISE) and polysomnography (PSG) tests was performed. Methods: Forty-five patients were included in the prospective study protocol. All patients enrolled in the study and underwent the following evaluations: (1) a drug-induced sleep endoscopy, with an evaluation of obstructions and collapse of the upper airways at 0° and in a HOBE position, with head and trunk elevation of 30°; (2) an overnight PSG assessment in the hospital with head and trunk elevation from 0° to 30° during the night; (3) a questionnaire to evaluate the feedback of patients to sleeping with head-of-bed elevation. Results: Velum (V) and oropharynx lateral wall (O) collapses were reduced in the 30° up position. There were no statistical differences that emerged in the obstruction of the tongue base and epiglottis between the 0° position and the 30° up position (*p* > 0.05). The average AHI score changed from 23.8 ± 13.3 (0° supine position) to 17.7 ± 12.4 (HOBE position), with a statistical difference (*p* = 0.03); the same statistical difference emerged in the percentage of apneas that decreased from 55 ± 28.1 to 44 ± 25.8 (*p* = 0.05). Conclusions: By adopting the HOBE position with 30° elevation of the head and trunk, it is possible to obtain a reduction of upper airways collapses and an improvement of apnea/hypopnea events and nightly respiratory outcomes.

## 1. Introduction

Obstructive sleep apnea (OSA) syndrome is one of the most common sleep-disorder breathing (SDB) conditions. It is characterized by the reduction (hypopnea) or complete cessation (apnea) of airflow in the upper airways during the night [1,2,3]. In some OSA patients, the frequency and duration of apneas could be influenced by head and body positions that are traditionally considered to be supine, prone, or lateral decubitus. Patients who present a reduction in the number or duration of apneas/hypopneas, related to changes in their sleeping position, have been classified as having position-dependent OSA (POSA) [4,5,6,7,8,9,10,11,12,13]. However, it should be considered that supine, prone, and lateral decubitus are not the only types of sleep positions possible. Head-of-bed elevation (HOBE), also known in clinical practice as Fowler’s position, is a standardized position in which the patient sleeps in a semi-sitting position (with a varying degree of bed–head elevation between 30° and 60°) [14,15]. This position allows a better patient chest expansion and improved breathing by facilitating blood oxygenation. It is a valid decubitus option used to improve breathing in a wide range of diseases (respiratory distress syndrome, chronic obstructive pulmonary disease, etc.) [14,15,16,17,18,19].

The aim of this prospective study was to evaluate the effectiveness of the HOBE position, with a 30° elevation of the head the trunk, in improving obstruction of the upper airways in OSA patients. A prospective trial simultaneously performing drug-induced sleep endoscopy (DISE) and polysomnography (PSG) tests was performed.

A 30° elevation of the head and trunk could be considered a good compromise between the possible effect of upper airway stabilization and patient tolerability to sleep in the HOBE position.

No studies evaluated the effect of 30° HOBE using a prospective protocol, with a PSG test in 30° upper position and a DISE in 30° upper position

## 2. Materials and Methods

### 2.1. Trial Design

All patients with a diagnosis of OSA consecutively referred to the Otolaryngology and Head Neck Department of the Morgagni Pierantoni hospital of Forlì, Italy, from January 2021 to January 2022, were initially considered as possible candidates for inclusion in the study.

**Figure 1:**
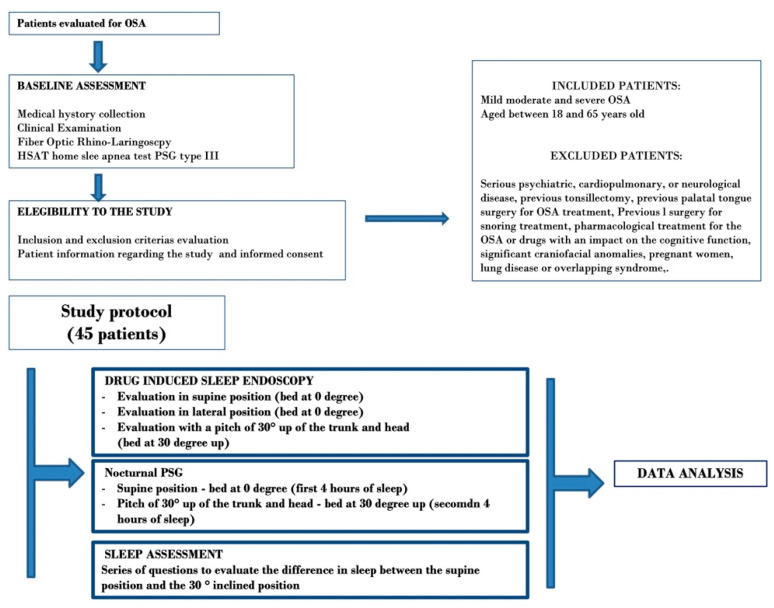
Flow chart of the study design.

The study design is reported in the flow chart of Figure 1.

At first, all patients underwent collection of medical history with BMI evaluation (clinical examination with a fiber optic rhino-laryngoscopy, as well as a type 3 polygraphy (HSAT: home sleep apnea testing). The sleep studies were carried out in an unattended way by means of a Polymesam Unattended 6-channel Device.

The apnea–hypopnea index (AHI) was collected to obtain the diagnosis of OSA and disease severity. Positional sleep apnea patients (POSA) were defined using Cartwright’s system (reduction of AHI >50% in the lateral decubitus).

Patients without OSA, with central or mixed apnea events or simple snoring, were excluded from the study.

OSA-diagnosed patients were subsequently screened for study inclusion, according to the inclusion and exclusion criteria reported in Figure 1.

### 2.2. Study Protocol

All patients enrolled in the study, according to the previous screening, underwent the following evaluations:

(1). A drug-induced sleep endoscopy (DISE), with evaluation of obstructions and collapse of the upper airways at 0° and in a HOBE position, with a head and trunk elevation of 30°.
(2). Overnight PSG assessment in the hospital, with head and trunk elevation from 0° to 30° during the night.
(3). Questions to evaluate the patients’ feedback to sleeping in HOBE position.

**Figure 2:**
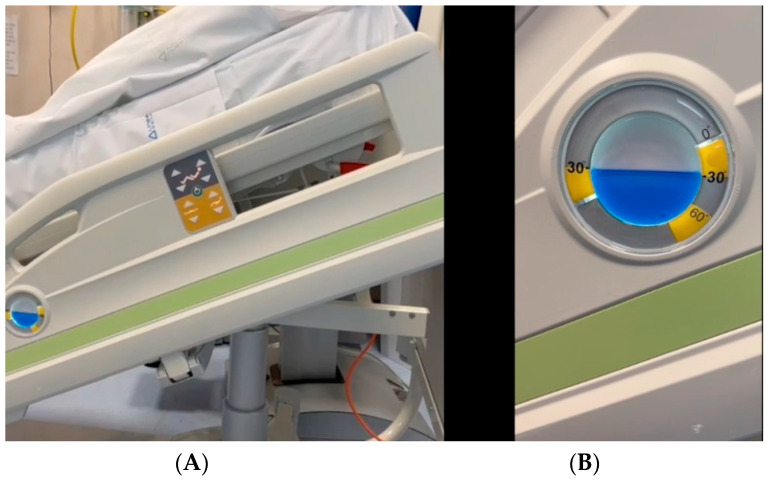
An anti-bedsore hospital bed used to conduct the clinical study. This type of bed offers the possibility, through a lateral remote control, to lift the bedhead up to a desired angle (**A**). With a specific protractor placed laterally to the bed, it is possible to estimate the achievement of the desired angle of the bedhead (**B**).

An anti-bedsore hospital bed was used to conduct this clinical study. This type of bed offers the possibility, through a lateral remote control, to lift the bedhead up to a desired angle. With a specific protractor, placed laterally to the bed, it was possible to estimate the achievement of the desired angle of the bedhead (Figure 2).

### 2.3. Drug Induced Sleep Endoscopy (DISE)

A DISE was performed on all enrolled patients, according to a standardized protocol (European position paper on DISE) [19]. Procedures were executed in the operating room with the anti-bedsore bed described above.

The VOTE system proposed by Kezirian et al. [20] was applied to classify all the DISE procedures.

**Figure 3:**
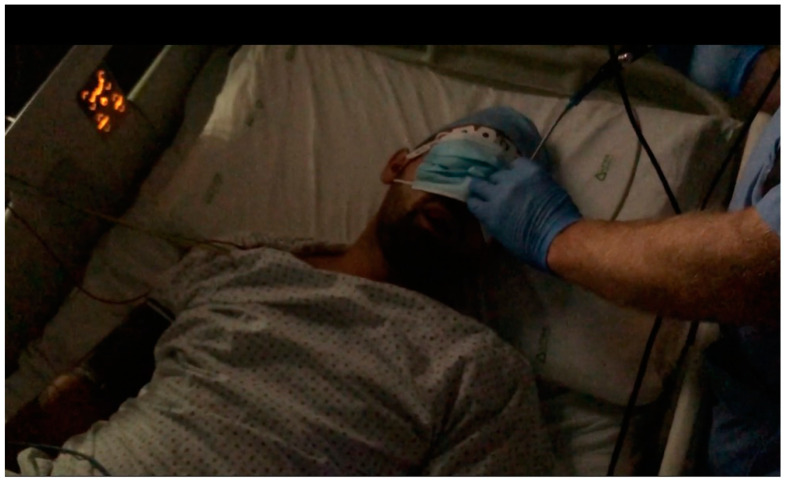
Head-of-bed elevation (HOBE) during drug-induced sleep endoscopy. Evaluation of possible changes in obstructive sites and collapses with the bedhead inclined 30° up.

After the evaluation of the upper airways in the supine position of 0°, for each patient, the bedhead was inclined up to 30° to examine possible modifications of the upper airway obstructions and collapses (HOBE position—Figure 3).

Changes of upper airway sites of obstruction and collapse in the passage from the supine position to the HOBE position were evaluated.

### 2.4. Polysomnography (PSG)

All the patients of the study underwent a nocturnal polysomnographic (PSG type III) examination in hospitalization mode, sleeping in the above-described inclinable bed.

The PSG test protocol was as follows: the patient was invited to go to sleep at 10:00 p.m. and wake up at 6:00 a.m. (Eight hours of sleep were recorded.) At 2:00 a.m., a nurse went to the patient’s bed, and without turning on the lights, changed the inclination of the bedhead, bringing it up to 30°. From this time, the sleep recording was conducted until 6 a.m. in the HOBE position (with an elevation of the patient’s head and trunk up to 30°). Four hours of sleep were performed in HOBE and non-HOBE positions, respectively. The study was repeated the following night if less than 8 h of sleep were recorded.

In all patients, PSG tests were scored by a blinded registered polysomnographic technician using established criteria. The PSG recording was divided into two parts of the night: the first part (22:00 p.m.–2:00 a.m.), during which the patient had slept lying in a supine position, and the second part (2:00–6:00 a.m.), in the HOBE position.

From each study part, data regarding the apnea–hypopnea index (AHI), oxygen desaturation index (ODI), lowest SpO2 (LOS), average SpO2, positional index, and CT90 were extracted and collected. The PSG data of each segment of the night were analyzed and compared.

### 2.5. Feedback of Patients

Feedback of patients to sleeping with head-of-bed elevation was investigated, with specific questions investigating if patients slept well or noted differences between the second part of the sleep in an elevated position and the first part in supine position. Finally, each patient was asked if he/she could sleep every night with a head-of-bed elevation.

### 2.6. Statistical Analysis

Nominal and continuous data were reported. To evaluate the differences between the two groups of patients the, χ^2^ test was employed. To compare the analyzed factors, the Student’s *t*-test was used. The normality of obtained data was tested before the use of the *t*-Student test. A threshold of *p* value < 0.05 was taken as the statistical significance. Statistical analysis was performed using the software SPSS (v26.0, IBM, Armonk, NY, USA).

## 3. Results

Initially, sixty-five patients were evaluated. Because of concomitant chronic obstructive pulmonary disease (10 cases), previous OSA surgical treatment (eight cases), and refusal of subjects (two cases), a total of twenty patients were excluded from the study.

Forty-five patients were included in the prospective study protocol.

The mean age of patients enrolled in the study was 46.4 years, with an s.d. of 14.5 years. The average BMI of the study group was 27.2 ± 3.1, while the average ESS was 8.3 ± 4.0.

Patient characteristics, comorbidities, and data of baseline PSG can be found in Table 1.

### 3.1. DISE Evaluation

From the evaluation of sites of obstruction and pattern of collapse between the supine position at 0° and HOBE at a 30° up position, interesting data emerged during DISE.

The results of the DISE evaluation, with the patient supine and in HOBE positions, is reported in Table 2.

In 82.3% of patients at 0° degree in supine position, a total collapse of the velum (V) and a subtotal collapse in 17.8% of cases was observed, whereas in the HOBE supine position, the group of study showed a total velum collapse in 57.7% of cases and a sub-total velum collapse in 28.8%. No collapse was evident in 13.3% of cases; this difference in the total velum collapse was statistically relevant (*p* = 0.02; see Table 2).

Total oropharynx lateral wall (O) collapse was present in 60% of patients in the supine 0° position. The incidence of total oropharynx lateral wall obstruction was reduced in the 30° up position in 33.3% of patients, with a statistical difference (*p* = 0.01).

No statistical differences emerged in the obstruction of tongue base and epiglottis between the 0° position and the 30° up position (*p* > 0.05 in all cases; see Table 2).

**Figure 4:**
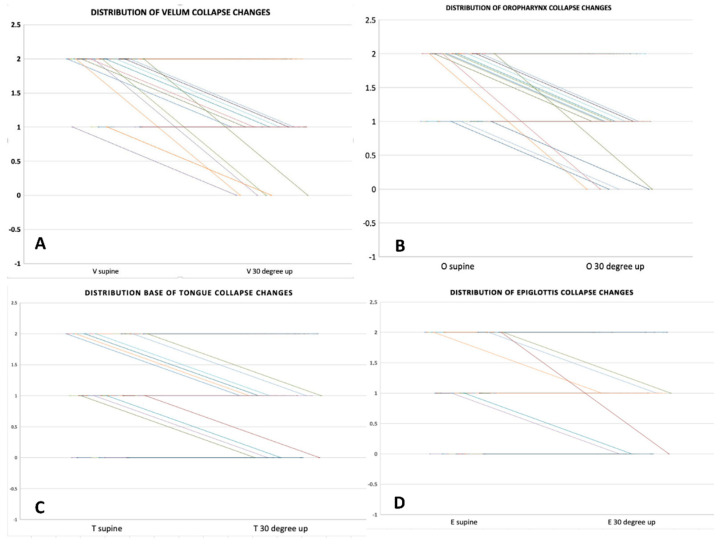
Distribution for each patient of changes in upper airway sites of obstruction related to position variation (from 0° to 30° up). (**A**) changes in velo-pharyngeal collapse; (**B**) changes in oropharyngeal collapse; (**C**) changes in base of tongue collapse; (**D**) changes in epiglottis collapse.

In short, the changes in DISE outcomes, passing from 0° to the HOBE 30° up position, were observed in 11 patients (24.4% of cases). No changes nor minor variations in grade, sites, and type of collapse were present in 75.6% of cases. The distribution of changes related to the grade of collapse (VOTE classification) in each patient at passage from 0° to 30° up is shown in Figure 4A–D.

### 3.2. PSG Outcomes

The PSG outcomes regarding the first part of the night (0°degree position) and the second part of the night (30° up position) are reported in Table 3.

The average AHI score changed from 23.8 ± 13.3 (0° supine position) to 17.7 ± 12.4 (HOBE position), with a significant statistical difference (*p* = 0.03); the percentage of apneas decreased from 55 ± 28.1 to 44 ± 25.8 (*p* = 0.05); average oxygen desaturation index (ODI) scores went down from 21.2 ± 10 to 16.1 ± 11.7 (*p*= 0.03); average SpO2 increased from 92 ± 3.3 to 93.7 ± 2.2 (*p* = 0.02). Similarly, the lower oxygen saturation scores rose from 83.4 ± 4.8 to 87.2 ± 3.1 (*p* = 0.0001). The only PSG parameter that did not show significant changes between the 0° and 30° up position was CT 90 (*p* = 0.1).

A positive effect on snoring was also recorded for the 30° up position. Snoring percentage went down from 17.3 ± 11.5 to 12.5 ± 12.6, with a statistical difference (*p* = 0.05).

### 3.3. BMI and 30° up Position

Study patients were sorted according to BMI classes. Within the group of 11 patients showing a positive effect of the 30° up position, five came from obesity class I and five from obesity class II, while just one patient was classified as pre-obesity. No patient belonging to the normal or underweight group showed a positive effect of the HOBE position.

Comparing pre-obesity vs. obesity class I and obesity class II, a statistical difference emerged (*p* < 0.05) by analyzing the distribution of patients with positive effects from the HOBE position. No statistical difference emerged for other class combinations (*p* > 0.05).

### 3.4. Lateral Position vs. 30° up Position

Following PSG results, 37% of patients of the study group were considered positional OSA, according to the Cartwright classification. Almost all the patients (10/11—90.9%) showing a positive effect of the 30° up position belonged to this group of POSA patients.

### 3.5. Patient Feedback

Patient feedback on supine sleep vs. 30° raised position is summarized in Table 4. This analysis revealed positive patient feedback regarding the night spent in the HOBE position.

## 4. Discussion

A limited number of physiological and clinical studies have been conducted to test the real effect of HOBE, compared to the supine position, in reducing OSA events, decreasing collapsibility of the mucous membranes, and increasing the upper airway area [12,15,16,17,18,21,22].

Souza et al. [11] have shown the effects of a mild degree of HOBE (7.5° elevation) on OSA severity and sleep quality. Fifty-two patients were evaluated. Compared to the baseline, HOBE significantly decreased the apnea–hypopnea index (AHI) from 15.7 to 10.7 events/h (*p* < 0.001) and increased minimum oxygen saturation from 83.5 to 87 (*p* = 0.003). The sleep architecture between the supine 0° position and HOBE results were very similar. However, sleep efficiency increased slightly but significantly with HOBE (87.2 vs. 88.8). In a similar way, McEvoy et al. [6] studied 13 male patients during the night and reported a reduction of the AHI from 49 ± 5 to 20 ± 7 events/h in the changing from supine to sitting position at 60°. Skinner et al. [17] reported a 22% reduction in AHI in 14 OSA patients when these patients were sleeping with a shoulder–head elevation pillow.

In contrast to the previous studies, Hsu et al. [15] employed DISE to evaluate the differences in upper airway obstruction sites and grade of collapse between the back-up head-elevated position (45° upward inclination) and the supine position: 198 patients underwent DISE first in the supine position and then in the HOBE position, with a 45° upward inclination.

A significant decrease in the velum anteroposterior collapse and velum concentric collapse (*p* < 0.001 and *p* < 0.001, respectively) was observed. The velum obstruction reduction was predominant in patients with mild OSA compared to moderate and severe OSA.

The objective of our prospective trial was to evaluate the effectiveness of the HOBE position (30° elevation) in reducing upper airways obstruction and apnea/hypopnea events in a group of OSA patients. No studies have evaluated the effect of 30° HOBE using a prospective protocol with a PSG test in 30°upper position and DISE in 30° upper position.

As illustrated above, the different degrees of head and trunk elevation have been described in literatures, ranging from 5° to 60°. Mild HOBE elevation (10–35°) is well accepted by the patients, whereas a 30° to 60° HOBE elevation is more poorly tolerated by patients in clinical practice. Therefore, we decided to test the effect of a 30° elevation of the head and trunk, considering this degree a good compromise between the possible effect of upper airway stabilization and patient tolerability while sleeping in the HOBE position.

During DISE evaluation, the presence of total velum collapse was reduced from 82.3% of cases in the 0° supine position to 57.7% of cases in the HOBE position (*p* = 0.02), and the incidence of total oropharynx lateral wall obstruction was reduced from 60% to 33.3% of cases (*p* = 0.01) using the HOBE position. On the other hand, there was no statistically significant improvement of collapse in the tongue base nor in the epiglottis when the patients were shifted from the supine into the 30° HOBE position. These results were in close agreement with those reported by Hsu et al.

In our study, the average AHI changed from 23.8 ± 13.3 (0° position) to 17.7 ± 12.4 (HOBE 30° position), with a significant statistical difference (*p* = 0.03); this AHI improvement of 6.1 events/hour was very similar to the data reported by Souza et al. (with an AHI improvement of 5 events/hour in the 30° HOBE position). Comparing the results of our study with the results of the studies described by McEvoy et al. (29 events/h) and Skinner et al. (22%), in which there was a greater improvement in AHI, we consider that this difference could be related to the higher degree of HOBE employed in these studies.

As a logical consequence of the decreased frequency of obstructive events in the 30° up HOBE position, all respiratory SpO2 parameters, except CT90, were improved.

What could the explanation be for the changes observed in upper airway collapsibility and blood saturation of OSAS patients when put in the HOBE position?

The pathophysiology of OSA is complex and is related to the interplay of both anatomical and non-anatomical factors, including neuromuscular responsiveness, ventilatory instability, and arousal threshold [19,20,21,22,23,24,25,26,27,28,29,30,31,32]. All these aspects should be considered in the genesis of POSA patients and could be related to the positive effect of the HOBE position.

Oropharyngeal sections seem to be increased in the Fowler position when compared with the supine one. In our setting, lifting the patient’s trunk by just 30° brought about an improvement of the antero-posterior diameter, as well of the lateral diameter. This data was in line with the physiological research reported by Souza et al. [11], which showed a significant increase in the mean upper airway volume, using a cervical computed tomography, from the supine to the head and trunk elevation position at 44° (40.35 ± 16.43 vs. 48.31 ± 16.21 cm^3^, respectively).

It is broadly accepted that in supine position during sleep, the tongue shows a frequent tendency to fall backwards, resulting in the obstruction of the upper airways. Despite a statistical difference, a collapse in the base of tongue did not show any improvement between the supine 0° and the decubitus 30° up during DISE evaluation. Concerning our study, it is possible to declare that the HOBE position may reduce the force of gravity of the tongue on the pharyngeal structures, leading to an improvement in the lateral and antero-posterior section collapse of the pharyngeal airway.

Another focal point to consider is derived from the diaphragm: the Fowler position stabilizes it in an inferior position, resulting in an abdominal pressure reduction, and at the same time, increases lung volume [27,28].

All the above discussed items are immediately effective after the change of position from decubitus in 0° to decubitus in the Fowler position. However, an additional but less immediately evident effect on pharyngeal anatomy and function is the so-called liquid rostral shift. Fowler posture seems to be effective in reducing liquid redistribution and its negative consequences on sleep breathing [27,28,29].

Finally, considering that all patients who showed a change related to the HOBE position were overweight or obese, it is possible to suggest that a higher BMI is related to worse lung expansion, due to the upward thrust of the diaphragm and increased abdominal pressure. This effect is reduced when patients assume the HOBE position [11,15,27].

Is the HOBE 30° position well-tolerated by OSA patients?

According to our results, 75.5% of patients tolerated well the HOBE position at 30° and furthermore, considered the idea of being able to sleep in this position every night without any problems. However, in some studies, with HOBE position greater than 35°, the authors reported a limited clinical applicability because patients did not tolerate sleeping with an excessive elevation of the head and trunk for long periods of time.

Finally, studies of the pathological and physiological effects of intermittent hypoxia in OSAHS identified two molecular pathways: a nuclear factor-κB (NF-κB)-dependent inflammatory pathway, which produces inflammatory cytokines, and a hypoxia-inducible factor-1 (HIF-1)-dependent adaptive pathway [30,31,32]. The possible effect of the HOBE position on these inflammatory patterns will be evaluated in future clinical studies.

### Strengths and Limitation

The prospective protocol for patient evaluation could be considered an advantage of this study because this is the first protocol combining the evaluation of a semi-sitting position effect on airway sections during DISE and sleep registration in the supine 0° vs. the semi-sitting position. The lower number of patients tested could be considered a limitation of the study. In addition, the PSG examination was split into two parts of the night (supine and HOBE position), which reduced recordings to 4 h for each of the two types of decubitus. However, in this regard, it should be noted that the PSG outcomes at a 0° supine position did not differ from the PSG report made prior to the enrollment of patients in the study.

## 5. Conclusions

By adopting the HOBE position with 30° elevation of the head and trunk, it is possible to obtain a reduction of upper airway collapses and an improvement of apnea/hypopnea events and nightly respiratory outcomes (based on average SpO2, lower SpO2).

## Figures and Tables

**Table 1:** Patient characteristics, comorbidities, and data of baseline PSG of the study group.

PATIENT’S FEATURES	
TOTAL PATIENTS 45	
Age	46.4 ± 14.5
Male	39 (86.6%)
Female	6 (13.3%)
BMI (average value)	27.2 ± 3.1
Epworth sleepiness scale (average value)	8.3 ± 4.0
Comorbidities	
Hypertension	12 (26.6%)
Diabetes	2 (4.4%)
Heart attack	1 (2.2)
Other cardiovascular comorbidities	3 (6.6%)
BASELINE PSG	
AHI	26.2 ± 9.9
Oxigen Desaturation Index (ODI)	24.5 ± 9.2
Average SpO2	91 ± 5.2
Lower Oxigen Saturation	81.9 ± 6.1
CT90	7.5 ± 3.6
Snoring percentage	19.3 ± 9.9
Positional patients according to lateral position (Cartwright classification)	17/45 (37%)

**Table 2:** DISE results according to 0° supine position and head-of-bed elevation (30° position).

DISE EVALUATION					
TOTAL PATIENTS 45					
	Supine with Bed 0° Degree		Supine Wit Bed 30° Elevated		*p*-Value (Chi Square Test)
	Number	%	Number	%	
Velum					
0 (no obstruction)	-	-	6	13.30%	0.02
1 (subtotal obstruction)	8	17.80%	13	28.80%	0.31
2 (total obstruction)	37	82.20%	26	57.70%	0.02
Oropharynx					
0 (no obstruction)	1	2.30%	6	13.30%	0.1
1 (subtotal obstruction)	17	37.70%	24	53.40%	0.2
2 (total obstruction)	27	60%	15	33.30%	0.01
Tongue base					
0 (no obstruction)	7	15.50%	11	24.40%	0.4
1 (subtotal obstruction)	20	44.50%	22	48.90%	0.8
2 (total obstruction)	18	40%	12	26.60%	0.2
Epiglottis					
0 (no obstruction)	25	55.50%	26	57.70%	>0.05
1 (subtotal obstruction)	11	24.50%	12	26.70%	>0.05
2 (total obstruction)	9	20%	7	15.50%	>0.05

**Table 3:** Differences in PSG outcomes between the 0° position and 30° up position.

PSG OUTCOMES			
	0 Degree	30 Degree	*p* Value Wilcoxon Signed-Rank Test Non Parametric Test
AHI (average value)	23.8 ± 13.3	17.7 ± 12.4	0.03
Percentage of Apneas	55 ± 28.1	44 ± 25.8	0.05
Percentage of Hypopneas	45 ± 28.7	56 ± 27.1	0.05
Oxigen Desaturation Index (ODI)	21.2 ± 10	16.1 ± 11.7	0.03
Average SpO2	92 ± 3.3	93.7 ± 2.2	0.02
Lower Oxigen Saturation	83.4 ± 4.8	87.2 ± 3.1	0.0001
CT90	6.5 ± 4.6	5.1 ± 3.4	0.1
Snoring percentage	17.3 ± 11.5	12.5 ± 12.6	0.05

**Table 4:** Feedback of patients to sleeping with head-of-bed elevation.

Patient’s Feedback TOTAL PATIENTS 45	YES Number	YES Percentage	NO Number	NO Percentage
Did you sleep well last night?	37	82.2%	8	17.8%
Have you noticed any differences between the second part of sleep in an elevated position and the first part in supine position?	10	22.2%	35	77.8%
Do you think that can sleep every night with a Head-of-bed elevation?	34	75.5%	11	24.4%

## Data Availability

The data presented in this study are available on request from the corresponding author.
